# Supplementary material for: Influence of Crosslinking Extent on Free Volumes of Silicone Rubber and Water Diffusion after Corona Discharge
Source: Materials (Basel). 2022 Oct 1;15(19):6833. doi: 10.3390/ma15196833 (PMC9571920; doi:10.3390/ma15196833)
Supplement: Supplementary file 1 [file materials-15-06833-s001.zip › materials-1915912-supplementary.pdf]

Supporting Information

Influence of Crosslinking Extent on Free Volumes of Silicone Rubber and Water Diffusion After Corona Discharge

Yue Yang [1], Zheng Wang [1,2], Xiangyang Peng [2], Zhen Huang [2], and Pengfei Fang [1,*]

[1] School of Physics and Technology, Wuhan University, Wuhan 430072, China
[2] Guangdong Key Laboratory of Electric Power Equipment Reliability, Electric Power Research Institute of Guangdong Power Grid Co., Ltd, Guangzhou 510080, China
* Correspondence: fangpf@whu.edu.cn

1. Corona discharge apparatus

A homemade needle-plate electrode system was applied in this study. As shown in **Figure S1**, the thickness of silicone rubber samples is 60±2 um. The material applied for manufacturing electrode is stainless steel. Diameter of the needle electrode is 3 mm and the curvature radius of the needle tip is 50±3 um. Diameter of the plate electrode is 20 mm and the thickness of it is 5 mm. The insulation sheet is made of silicone rubber. The purpose of placing an insulation sheet is to prevent short circuit. During the corona discharge process, 10 kV AC voltage was applied to the needle electrode and the plate electrode is grounded. The distance between sample surface with needle tip is 3 mm. The corona discharge aging apparatus was designed for simulating serving environment of composite insulators, so the frequency of AC voltage is 50 Hz. The electric field strength distribution of the system under 10 kV voltage at a transient is shown in **Figure S2**. The electric field is strongest near the needle tip and decays very fast as the distance away from the needle tip grows, as shown in **Figure S3**.

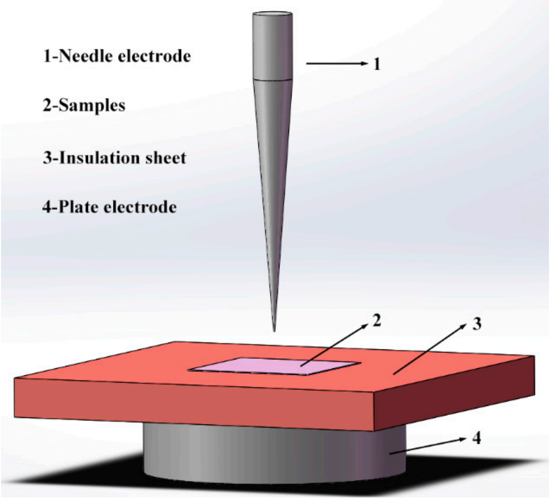

Figure S1 Schematic diagram of needle-plate electrode system

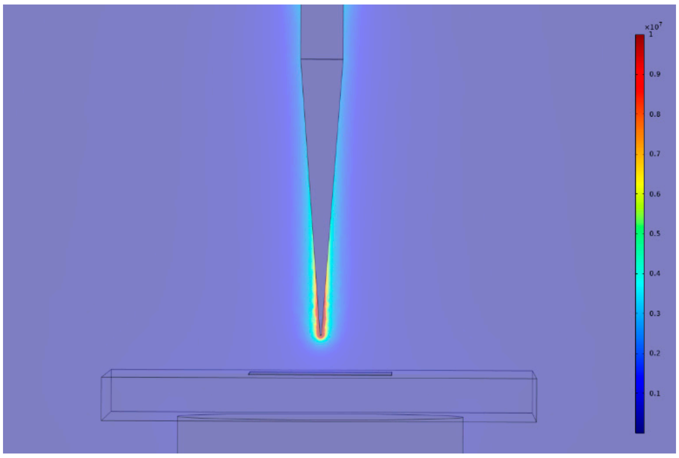

Figure S2 Electric field strength distribution under 10 kV voltage

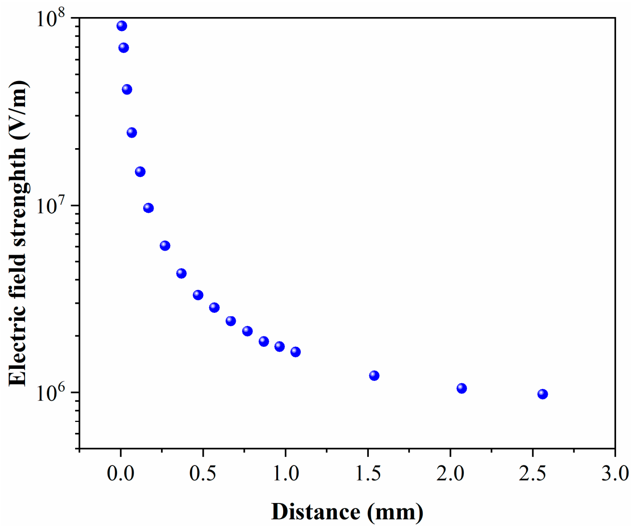

Figure S3 The electric field strength as a function of distance from the needle tip to the sample surface

The breakdown field of air is 3 kV/mm under standard atmospheric pressure at room temperature. As shown in **Error! Reference source not found.**, the electric field strength below 3×10^7 V/m at the position very near to the needle tip and decreases sharply to 10^6 V/m. The strong electric field near the needle tip can trigger ionization of air and generate plasma discharge (corona discharge is one kind of plasma discharge) under AC voltage. The results indicate that the corona discharge phenomenon in this study is plasma discharge in air but not streamer discharge.

2. Electrochemical impedance measurement apparatus

A three-electrode measurement system was applied in this study, as shown in **Figure S4**. The sample was placed on the surface of ITO glass and a plastic tube was sealed on the sample vertically. The ITO glass is served as working electrode and the sample. A platinum plate

and a saturated calomel electrode are served as counter electrode and reference electrode, respectively.

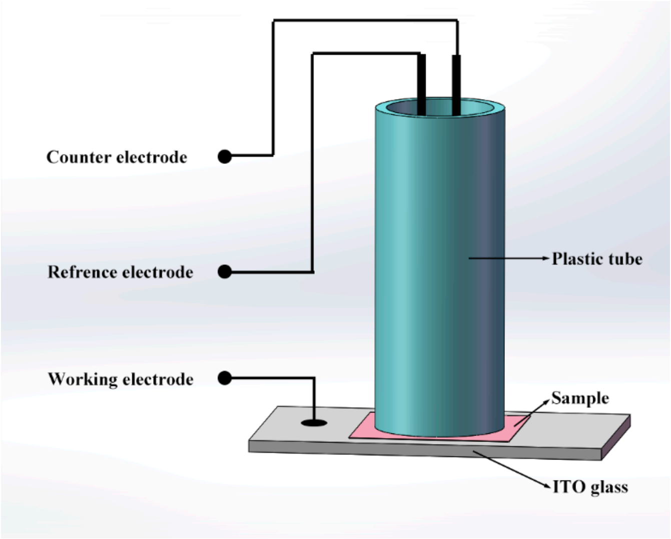

Figure S4 Schematic diagrams of EIS measurement system

3. Chemical structure and morphology of samples before corona

The FTIR spectrum of silicone rubber sample with diverse cross-linker weight ratio are shown in **Figure S5** and the correspondence between wavenumber with characteristic chemical groups in silicone rubber are shown in

Table S1. The intensity and peak position do not vary with the increase of cross-linker weight ratio, as shown in **Figure S5**.

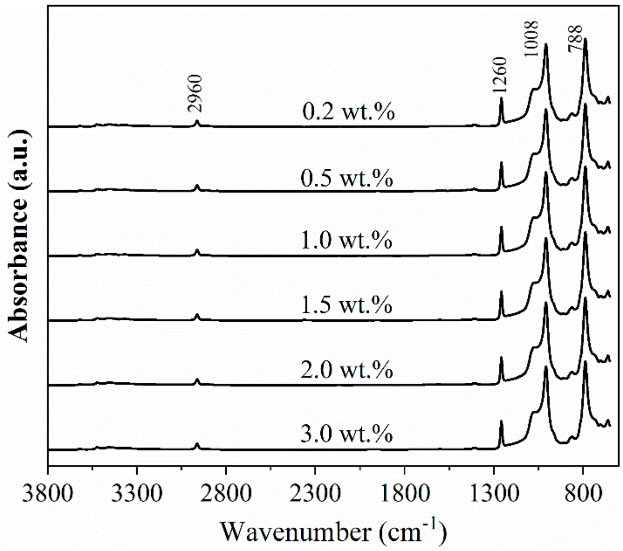

Figure S5 FTIR spectrum of silicone rubber with diverse cross-linker weight ratio before corona treatment

Table S1 Corresponding wavenumber to characteristic chemical groups of silicone rubber

Wavenumber (cm^{-1})	Chemical groups
2960	$-CH_3$
1260	$Si-CH_2$
1008	Si-O-Si
788	$-CH_3$

The SEM spectrum of silicone rubber before corona discharge treatment are shown in **Figure S6**. All the samples have a smooth surface with few inorganic filler particles can be observed. The results indicate that the micro surface mophorlogy of silicone rubber is not influenced by cross-linker weight ratio.

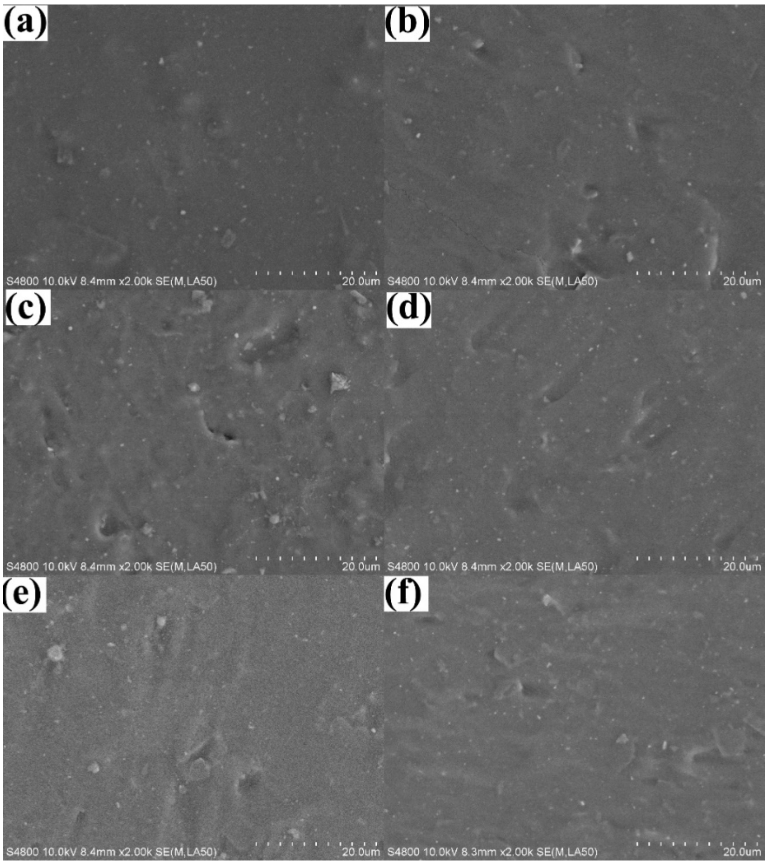

Figure S6 SEM images of silicone rubber before corona discharge with diverse cross-linker weight ratio: 0.2 wt.%(a), 0.5 wt.%(b), 1.0 wt.%(c), 1.5 wt.%(d), 2.0 wt.%(e) and 3.0 wt.%(f), respectively.
